# A pilot study of leakage and compartmentalization of the contrast agent Ablavar

**DOI:** 10.1186/1532-429X-15-S1-E7

**Published:** 2013-01-30

**Authors:** Octavia Bane, Daniel C Lee, Brandon Benefield, Michael Markl, James Carr, Timothy J Carroll

**Affiliations:** 1 Radiology, Northwestern University, Chicago, IL, USA; 2 Biomedical Engineering, Northwestern University, Evanston, IL, USA; 3 Feinberg Cardiovascular Institute, Northwestern University, Chicago, IL, USA

## Background

We evaluate the compartmentalization of the blood pool agent Ablavar (Lantheus Medical Imaging) for the quantification of steady-state tissue blood volume.

## Methods

Simulation study: In vitro studies have shown Ablavar to be 80-90% bound to albumin, with up to 10 fold relaxivity difference between bound and free fractions. We performed simulations to assess the effect of extravasation of the free fraction on signal. Vascular fraction measurements were simulated assuming slow two-compartment exchange for different contrast agent injection concentrations, binding fractions, bound and free relaxivity, and true vascular fractions.

Volunteer study: five healthy volunteers (4 males, average age 33) underwent T1 measurement pre and 2 minutes post administration of five injections of 0.006 mmol/kg (a fifth of a single dose) Ablavar. Steady-state T1 was mapped using a cardiac gated Modified Look Locker Inversion Recovery (MOLLI) pulse sequence (slice thickness 8 mm, FOV 300 x 400 mm^2^, matrix 256 x 172, effective TI 100 ms).

Image Processing: Maps of vascular fraction were calculated from signal difference maps, according to a slow water exchange model. Fv was measured in the myocardium, dome of the liver, and skeletal muscle visible on the short axis MOLLI images, and was corrected for Ablavar extravasation based on the leakage study.

The true fv and exchange rate of water protons was determined by chi square minimization between data and simulations of the effect of water exchange on fv according to the two compartment water exchange model presented by Donahue* et al.* (1996).

## Results

**Figure 1:**
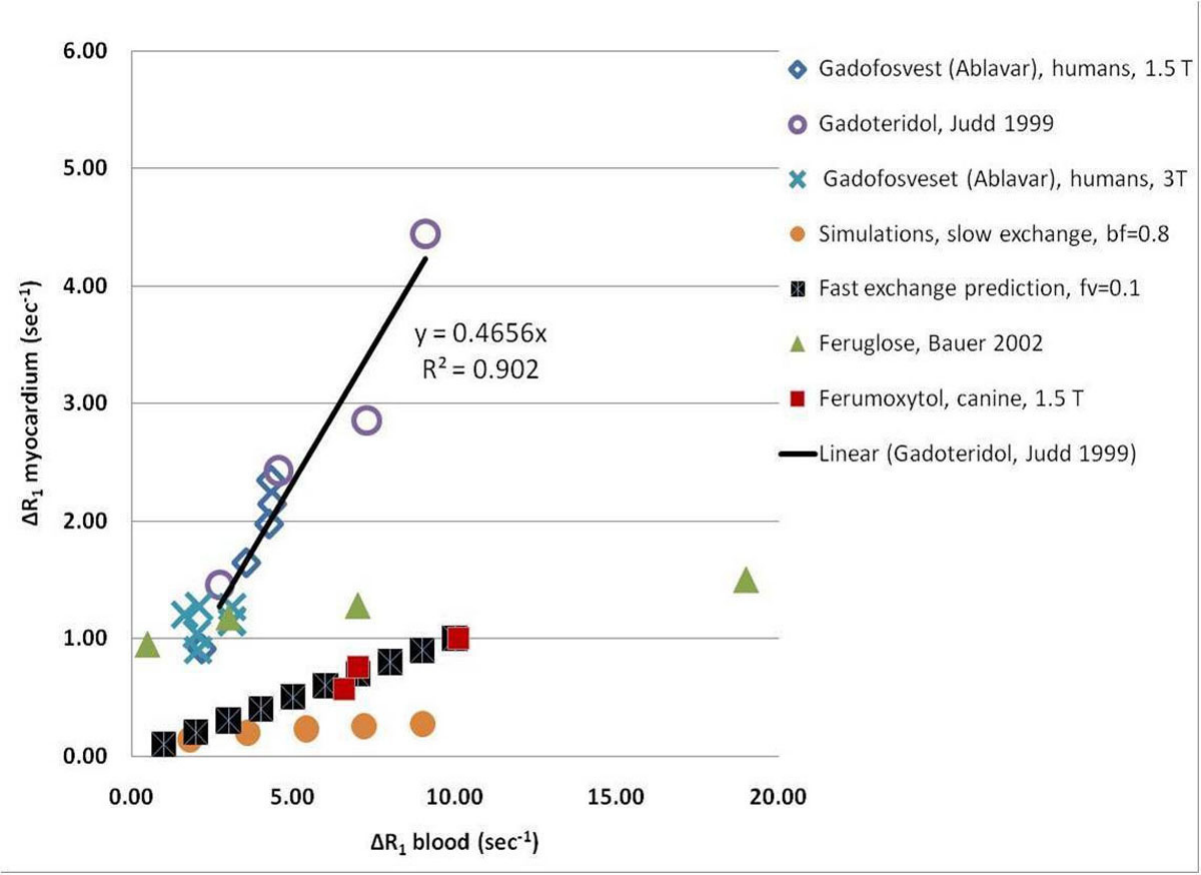
Myocardium longitudinal relaxation rates R1 as a function of blood longitudinal relaxation rates for Ablavar in healthy volunteers displays the same linear relationship as an extracellular agent. In comparison, intravascular USPIO agents feruglose and ferumoxytol display a non-linear trend. Ferumoxytol data is within the bounds predicted by the slow and fast exchange limits of the two compartment model.

We found that the effect of partial binding of Ablavar on the measurement of vascular fraction is less that 20%. The true vascular fractions and exchange rates are summarized in Table 1. A comparison of the myocardium relaxation rate induced by administration of Ablavar in healthy volunteers imaged at 1.5T and 3T, with other extracellular and intravascular contrast agents shows that Ablavar behaves like an extracellular contrast agent (Figure 1).

**Table 1:** Measurements of vascular fraction

	Measured fv (%)	Measured exchange rate (Hz)	Fv slow exchange (%) Donahue *et al.*	Fv fast exchange Donahue *et al.*
Myocardium	25	41	-	-
Liver	45	3.7	41± 6	26±7
Muscle	16	1	8±3	15±1

Measurements of vascular fraction compared to those of Donahue *et al.* (1996) in the fast and slow limits of the water compartmentalization model.

## Conclusions

The measured values of fv in liver and muscle agree with the Donahue model. Measured myocardial fv values over-estimate published values (9-12%), and approach those of extracellular volume (25%), which suggests the intravascular assumption may not be appropriate for Ablavar. The distribution of the volunteer data indicates that a three-compartment model, with slow exchange of Ablavar and water protons between the vascular and interstitial compartments, and fast water exchange between the interstitium and the cells is required to use Ablavar for quantification of blood volume.

## Funding

R01 NHLBI HL088437

Lantheus Medical Imaging provided the agent

